# From Fly to Human: Translational Relevance of *Drosophila* Models in the Study of Vitamin B6 and Cancer Relationship

**DOI:** 10.3390/ijms27062877

**Published:** 2026-03-22

**Authors:** Fiammetta Vernì, Chiara Angioli, Angelo Ferriero, Beatrice Agostini

**Affiliations:** Department of Biology and Biotechnology “Charles Darwin”, Sapienza University, 00185 Rome, Italy

**Keywords:** vitamin B6, pyridoxal 5′-phosphate (PLP), cancer, DNA damage, chromosome aberrations (CABs), reactive oxygen species (ROS), serine hydroxymethyltransferase (SHMT), *Drosophila*

## Abstract

Vitamin B6 is an essential micronutrient whose biologically active form, pyridoxal 5′-phosphate (PLP), acts as a cofactor in metabolic reactions linked to tumorigenesis and also functions as an antioxidant. Low plasma PLP levels are consistently associated with cancer, but studies on dietary intake have yielded conflicting results. Overall, evidence suggests that the effects of vitamin B6 deficiency on cancer are context-dependent, varying with cell type and tumor stage. Accordingly, high expression of *PDXK* and *PNPO*, two key genes involved in PLP biosynthesis, is associated with tumor progression in some malignancies, whereas it correlates with improved outcomes in others. This review explores *Drosophila melanogaster* as a useful model to investigate underlying mechanisms, bypassing the limitations of human studies. Research in *Drosophila* demonstrates that PLP deficiency promotes cancer by triggering genomic instability. Furthermore, a critical PLP-*SHMT* gene–nutrient interaction impacting oncogenesis has been established in flies, offering significant therapeutic implications. Finally, studies in *Drosophila* have shown that PLP deficiency can promote tumor development by also triggering the loss of heterozygosity (LOH). These findings highlight *Drosophila* as a powerful tool to elucidate the molecular pathways linking vitamin B6 deficiency to cancer.

## 1. Vitamin B6

Vitamin B6 is a generic term used to indicate a group of six interconvertible water-soluble chemical compounds (vitamers) all containing a pyridine ring in the center: pyridoxine (PN), pyridoxamine (PM), pyridoxal (PL), and the respective 5′-phosphorylated forms (PNP, PMP, PLP). The chemical formula of vitamin B6 was initially reported by Ohdake in 1932 [1]. In 1938, crystalline vitamin B6 was isolated from yeast by five independent research groups. Following the characterization of its structure one year later, György named the vitamer “pyridoxine” (PN) to denote its structural homology with the pyridine ring [2]. In the same year, the total synthesis of vitamin B6 was successfully achieved by Harris and Folkers [3].

PLP represents the biologically active form, functioning as a coenzyme in many enzymatic reactions, including decarboxylation, deamination, transamination and racemization. However, PMP has also been shown to play a catalytic role in specific reactions [4].

**Figure 1:**
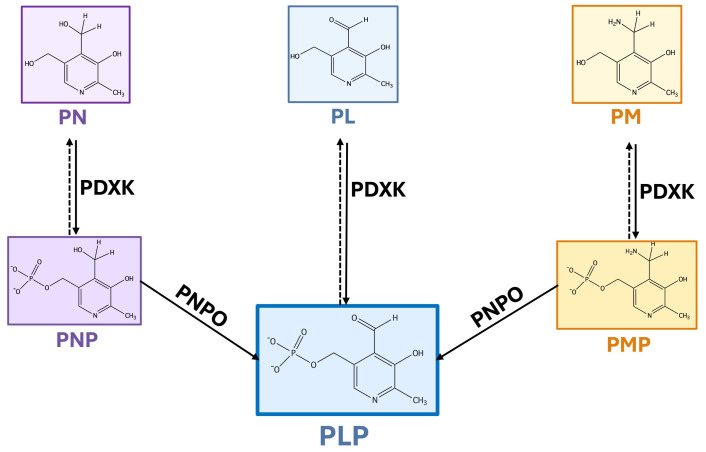
Vitamin B6 salvage pathway. The diagram illustrates the interconversion of vitamin B6 vitamers through the salvage pathway. The non-phosphorylated forms—pyridoxine (PN), pyridoxal (PL), and pyridoxamine (PM)—are converted into their respective phosphorylated derivatives—PNP, PLP, and PMP—by the enzyme pyridoxal kinase (PDXK). Subsequently, pyridoxine 5′-phosphate oxidase (PNPO) catalyzes the oxidation of PNP and the deamination of PMP to generate pyridoxal 5′-phosphate (PLP), the primary biologically active cofactor. Dashed arrows indicate the reversible dephosphorylation reactions mediated by phosphatases.

Unlike plants and microorganisms, which synthesize vitamin B6 de novo, humans acquire it from external sources and utilize a salvage pathway to interconvert the different vitamers [5]. In the salvage pathway (Figure 1), PL, PN and PM are first phosphorylated by a single ATP-dependent pyridoxal kinase (PDXK or PLK), and then oxidized to PLP by a FMN-dependent pyridoxine/pyridoxamine 5′-phosphate oxidase (PNPO) [6].

After ingestion, B6 vitamers are hydrolyzed by intestinal phosphatases into PL, PM, and PN, which are then absorbed and transported to the liver, where PDXK phosphorylates them. PNPO, subsequently, catalyzes the oxidation of PNP and PMP into the active PLP form. PLP is distributed through the blood bound to albumin, but it must be converted back to PL by nonspecific alkaline phosphatases (TNSALP) to cross cell membranes. Once inside the cell, PDXK restores the phosphate group, allowing the PLP to bind to apo-B6 enzymes [7].

The 4′-aldehyde group of PLP is highly reactive and can trigger harmful non-specific reactions if its levels become excessive. Thus, the maintenance of appropriate intracellular PLP levels is essential. Cells regulate this balance through various mechanisms including product inhibition, a process in which PLP itself inhibits its own production by acting on PDXK and PNPO. Additionally, pyridoxal phosphatases fine-tune the overall concentration [8]. Recently, the highly conserved PLP-binding protein (PLP-BP) has emerged as a key player in vitamin B6 homeostasis across various organisms. While traditionally considered a chaperone that protects PLP from degradation and delivers it to apoenzymes, this hypothesis is currently under debate. Emerging evidence suggests a novel role for PLP-BP as an RNA-binding protein involved in the post-transcriptional regulation of gene expression [9].

Knowledge regarding PLP catabolism in humans and other mammals remains limited. The predominant end-product of vitamin B6 degradation is 4-pyridoxic acid (4-PA), which is eliminated via urinary excretion. The synthesis of 4-PA occurs through a two-stage process: initially, the intracellular enzyme PLP phosphatase (PLPase) dephosphorylates PLP into PL; subsequently, PL undergoes oxidation to 4-PA, a reaction catalyzed by either a non-specific aldehyde oxidase (AOX) or aldehyde dehydrogenase (ALDH) [10].

### 1.1. Vitamin B6 Roles

PLP serves as an essential cofactor for numerous enzymes that collectively catalyze approximately 4% of all cellular metabolic reactions [5]. These enzymes are pivotal in fatty acid biosynthesis, glycogen degradation, and the synthesis of tetrapyrroles including heme, cobalamin, and chlorophyll [11]. Furthermore, PLP is indispensable for the production of key neurotransmitters such as epinephrine, dopamine, serotonin, and GABA; additionally, it plays a structural role in the proper folding of PLP-dependent apoenzymes [11,12]. Beyond catalysis, PLP modulates steroid hormone receptor activity and immune function [13,14]. Notably, B6 vitamers exhibit protective properties: PLP and PMP counteract the formation of genotoxic advanced glycation end products (AGEs), while all vitamers can quench reactive oxygen species (ROS) through their hydroxyl and amine ring substituents [7,15]. Additionally, PLP mitigates oxidative stress by acting as a cofactor in the synthesis of cysteine, the rate-limiting precursor for glutathione, one of the major cellular antioxidants [16].

### 1.2. Causes and Consequences of Vitamin B6 Deficiency

Based on current guidelines, the Recommended Dietary Allowance (RDA) for vitamin B6 is 1.3 mg daily for adults aged 19 to 50. For those aged 51 and older, the requirement increases to 1.7 mg/day for men and 1.5 mg/day for women. Vitamin B6 is a widely distributed nutrient present in both animal- and plant-derived foods. The richest and most easily absorbed sources are found in meat and fish, particularly beef liver, chicken, turkey, and fish such as tuna and salmon. Among plant-based foods, chickpeas are an exceptionally high source of this vitamin. Significant amounts are also present in potatoes. Fruits also contribute to daily intake; bananas are the most common and well-known source, followed by tropical fruits like papaya. Finally, many common items, such as breakfast cereals, are often fortified with vitamin B6 to help individuals easily reach their recommended daily allowance [16].

While primary vitamin B6 deficiency is uncommon due to its widespread availability in various food groups, secondary deficiencies frequently arise from malabsorption, genetic predispositions, increased physiological demand, or drug interactions. Depleted PLP levels are often associated with renal dysfunction and gastrointestinal disorders, such as celiac and inflammatory bowel diseases [17]. Furthermore, subclinical deficiencies are prevalent in cases of alcohol dependence, pregnancy, obesity and diabetes [18,19,20]. Pharmacological agents like isoniazid and penicillamine can also impair PLP availability [21], while mutations in the *PDXK* and *PNPO* genes lead to severe neurological manifestations [16]. Given its pleiotropic nature, B6 deficiency manifests a broad clinical spectrum, including anemia, immune impairment, and metabolic disorders. Consequently, vitamin B6 is increasingly implicated in the pathogenesis of diabetes, different types of cancer, and neurological diseases, although the precise molecular mechanisms remain to be fully elucidated [16,20,22]. Notably, excessive vitamin B6 intake, most commonly due to inappropriate high-dose supplement use, can cause peripheral neurotoxicity, particularly with chronic exposure. Symptoms usually improve after discontinuation but may persist in some individuals [23].

### 1.3. Vitamin B6 and Cancer

Since the 1950s, preclinical research has explored how variations in vitamin B6 availability influence cancer growth. In vitro and animal studies revealed that vitamin B6 deficiency could exert antineoplastic effects by slowing tumor growth [24,25]. This effect was attributed to the hypothesis that malignant cells, characterized by accelerated metabolism, require elevated PLP levels to sustain their proliferation [26,27]. Several attempts were therefore made to use B6 antagonists, such as 4-deoxypyridoxine (4-DP), as antitumor agents to reduce PLP levels; however, their clinical translation was limited by systemic toxicity [28,29].

Although a few studies indicated that vitamin B6 deficiency could promote cancer growth [30] and early clinical findings in the 1960s disproved the idea that B6 deficiency would exert antineoplastic effects [31], the prevailing view until the early 1980s remained that vitamin B6 restriction was beneficial in tumor treatment by counteracting the accelerated PLP metabolism and reducing cell proliferation [32,33]. Conversely, starting in the mid-1980s, researchers began to explore the alternative hypothesis that vitamin B6 may possess antineoplastic properties, at least under specific conditions. Since then, a body of evidence has emerged demonstrating that vitamin B6 administration can inhibit tumor proliferation. It was shown that the administration of vitamin B6 vitamers, specifically PN or PL, was able to arrest the growth of cancer cell lines such as rat hepatoma [34] and human and murine melanoma cells [35,36,37]. Additionally, in vivo studies demonstrated that the injection or dietary supplementation of vitamin B6 vitamers in tumor-bearing mice suppressed neoplastic growth [38,39,40,41].

Despite this evidence, the use of vitamin B6 to prevent cancer recurrence has yielded inconsistent results. Early reports from 1977 and subsequent trials involving vitamin cocktails suggested that B6 could reduce the recurrence rate of bladder cancer [42,43], but other clinical trials have failed to replicate these findings [44].

During the 1980s, accumulating evidence demonstrated that PLP levels decrease along with tumor progression [22]. Specifically, an inverse correlation was observed between the progression of experimental hepatomas and the intratumoral levels and bioavailability of PLP [45,46]. In addition, studies on colorectal cancer (CRC) revealed that colon adenocarcinoma samples exhibited vitamin levels significantly higher than those in surrounding healthy colon tissue. In contrast, vitamin B6 was found to be significantly depleted in hepatic CRC metastases compared with normal liver tissue [47]. To explain the progressive depletion of vitamin B6 during cancer progression, it was proposed that the immune system plays a crucial role by sequestering vitamin B6 to support its own metabolic requirements, thereby limiting its availability to tumor cells [48,49]. Consistently, the immunological status of immunocompromised cancer patients was notably improved through PN supplementation [50,51].

**Figure 2:**
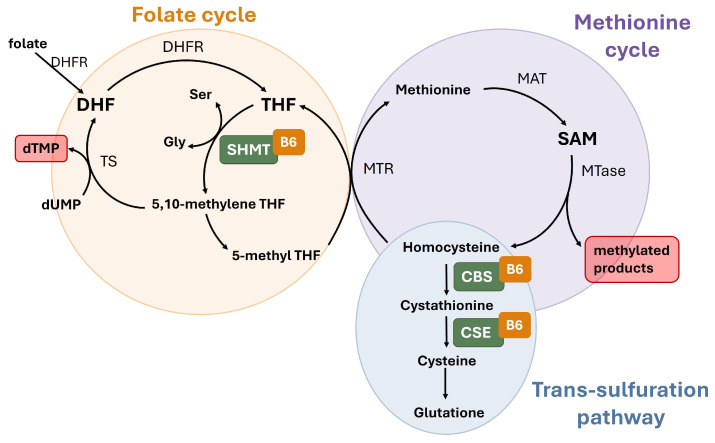
One carbon metabolism. Folate cycle (**left**) facilitates the synthesis of dTMP from dUMP via Thymidylate Synthase (TS) and the interconversion of THF derivatives. A critical step is mediated by the vitamin B6-dependent enzyme Serine Hydroxymethyltransferase (SHMT), which converts serine and THF into glycine and 5,10-methylene THF. In the methionine cycle (**right**), Methionine synthase (MTR) regenerates methionine from homocysteine. Methionine is subsequently converted to S-adenosylmethionine (SAM), the primary universal methyl donor for methyltransferases to generate methylated products (DNA, proteins, and lipids). Excess homocysteine can be diverted into the Trans-sulfuration pathway (**bottom**), where the vitamin B6-dependent enzymes Cystathionine beta synthase (CBS) and Cystathionine gamma-lyase (CSE) catalyze the synthesis of cysteine and the antioxidant Glutathione (GSH).

More recently, the role of vitamin B6 within one-carbon (1C) metabolism has gained considerable interest in the context of cancer development. In this pathway (Figure 2), PLP serves as an essential cofactor for key enzymes such as serine hydroxymethyltransferase (SHMT), which drives nucleotide synthesis and methyl group supply, as well as for those in the trans-sulfuration pathway, which are critical for glutathione production [52]. This central biochemical role has led to the hypothesis that optimal PLP levels are required to maintain genomic integrity, ensure proper methylation patterns, and support cellular antioxidant defenses.

Starting from this hypothesis, several retrospective and prospective studies have been conducted to evaluate the correlation between dietary vitamin B6 intake and plasmatic PLP levels with cancer risk. Beyond circulating PLP as a standard biomarker, several studies have also employed the PAr index (the ratio of 4-pyridoxic acid to the sum of pyridoxal and pyridoxal-5′-phosphate) as a more sensitive indicator of accelerated vitamin B6 catabolism [53].

With few exceptions, these investigations consistently identified an inverse correlation between vitamin B6 status and the risk and/or survival outcomes for several malignancies [22].

Large-scale prospective studies have highlighted a significant link between B6 status and lung cancer risk. Data from a cohort of approximately 520,000 participants recruited by The European Prospective Investigation into Cancer and Nutrition (EPIC) revealed that serum levels of vitamin B6 and methionine are inversely associated with lung cancer risk. This association remained significant regardless of smoking status [54]. Similarly, results from the Lung Cancer Cohort Consortium, encompassing 20 prospective cohorts across four continents, confirmed that reduced serum PLP levels, alongside an increased PAr index, are associated with a higher risk of developing lung cancer. This suggests that increased vitamin B6 degradation, driven by inflammation and immune activation, may play a crucial role in lung carcinogenesis [55]. A dose-dependent relationship has also been established between circulating PLP levels and pancreatic cancer risk. A meta-analysis of observational studies concluded that high blood concentrations of PLP may exert a protective effect against the development of this malignancy [56]. In a case–control study nested in the Multiethnic Cohort in Hawaii and Southern California elevated circulating PLP levels have been found to protect against invasive postmenopausal breast cancer, with a more pronounced effect observed in hormone receptor-positive (ER+/PR+) tumors [57].

The association between PLP and cancer is particularly robust in CRC. Multiple prospective nested case–control studies, including the Nurses’ Health Study [58], the Physicians’ Health Study [59] and a multiethnic cohort [60] have consistently reported an inverse association between plasma PLP concentrations and the incidence of this malignancy. Notably, Lee et al. [59] concluded that vitamin B6 exerts a protective effect against CRC independently of other one-carbon metabolites and inflammatory biomarkers. In a large, population-based study, the 3-hydroxykynurenine: xanthurenic acid (HK:XA) ratio was evaluated alongside circulating PLP levels and the PAr index to assess functional vitamin B6 status. Higher HK:XA ratios (reflecting lower functional status) and elevated PAr levels were both significantly linked to increased CRC risk, and these associations were more consistent with a role in tumor progression rather than initiation [61]. These findings were further corroborated by a large Chinese hospital-based study which showed that while PLP levels were inversely associated with cancer risk, the PAr index exhibited a positive correlation, supporting the involvement of inflammation and oxidative stress in colorectal carcinogenesis [62]. Beyond its preventive role, preoperative vitamin B6 status has been reported as a significant prognostic indicator, with higher levels correlating with improved overall survival in patients with stage I–III CRC [63].

While circulating PLP levels consistently correlate with reduced cancer risk, studies on dietary vitamin B6 intake have shown less consistency. Early meta-analyses and cohort studies, such as those by Larsson et al. and Zhang et al., refs. [64,65] reported a lack of association or noted significant heterogeneity among findings. Zhang et al. specifically suggested that results from adult cohort studies may be inconsistent when conducted within relatively well-nourished populations, in which baseline intake is already sufficient to satisfy biological requirements.

A comprehensive systematic review by Mocellin (2017) [66], covering both observational and intervention studies, revealed a robust inverse association between higher PLP levels and gastrointestinal tumors, but the evidence for dietary intake was less consistent. A favorable correlation was observed for food-derived B6, but not when supplements were included in the total intake.

In contrast, recent evidence provides more consistent results. A meta-analysis by Lai et al. (2023) [67], encompassing 20 cohort and 8 case–control studies, reported a statistically robust association between both vitamin B6 intake and blood PLP levels with a reduced risk of CRC. The authors noted that incorporating recent large-scale data [68,69] increased the statistical power of their analysis. This enhanced sensitivity enabled the identification of significant outcomes that had remained elusive in earlier, smaller-scale analyses. These findings have also expanded the potential protective role of vitamin B6 beyond gastrointestinal sites, suggesting, for instance, a possible reduction in ovarian cancer risk associated with higher dietary intake [68].

Discrepancies across these studies may reflect the challenge of establishing a clear causal link between micronutrient intake and cancer. This is largely due to confounding factors, as high vitamin consumption often correlates with overall healthy behaviors that independently reduce cancer risk, making it difficult to isolate the specific effect of vitamin B6. Moreover, since nutrients are consumed together in complex foods, it is often hard to disentangle the effect of a single vitamin and to identify which specific component is providing protection. Additionally, chronic inflammation and genetic variability can alter vitamin bioavailability and metabolism, leading to a discrepancy between dietary intake and systemic levels.

### 1.4. Expression of Vitamin B6 Salvage Pathway Genes in Cancer

Consistent with the notion that metabolic reprogramming of cancer cells requires an increased supply of cofactors to support accelerated biosynthetic pathways, recent evidence highlights the dysregulation of the vitamin B6 salvage pathway as a critical feature in various malignancies. The two key enzymes of this pathway, PDXK and PNPO (Figure 1), often show altered expression levels that correlate with tumor progression and patient prognosis. However, the expression patterns of these genes vary markedly across cancer types, being either upregulated or downregulated depending on the context. In lung cancer, higher *PDXK* levels have been associated with a more favorable outcome by restoring PLP levels, which otherwise decline progressively [70]. Conversely, *PDXK* was found to be highly expressed in acute myeloid leukemia (AML) blasts, likely to satisfy their increased nucleotide requirements to sustain rapid proliferation [71]. In these cells, decreasing PDXK activity or using PLP inhibitors reduces cancer growth.

*PDXK* is also upregulated in serous ovarian cancer cells, where high expression levels are associated with poor prognosis [72]. In hepatocellular carcinoma cell lines, upregulation of *PDXK* promotes proliferation and metastasis, suggesting that *PDXK* expression may serve as a potential diagnostic and therapeutic target [73].

*PNPO* was included in a panel of seven genes that can predict the overall survival of CRC patients [74]. Additionally, *PNPO* overexpression has been associated with epithelial ovarian cancer (EOC); accordingly, *PNPO* knockdown was found to decrease cancer proliferation and invasiveness [75]. The suppression of *PNPO* in breast infiltrating ductal carcinoma inhibits cell proliferation and migration and induces apoptosis [76]. Furthermore, computational analyses have shown that at least 21 tumor types overexpress *PNPO* at both mRNA and protein levels, which is often prognostically significant. Genomic studies have revealed that the *PNPO* gene is altered in about 1.3% of all tumors. In most cases, the *PNPO* gene is amplified, although missense, truncating mutations, or deep deletions have also been observed [77].

Interestingly, *PNPO* overexpression is more frequently associated with cancer development than *PDXK* overexpression. It is tempting to speculate that this reflects PNPO’s role as the rate-limiting enzyme in PLP synthesis. While PDXK performs the initial phosphorylation, PNPO controls the final, critical oxidation step that converts the primary accumulated vitamin B6 intermediates (PNP/PMP) into the active cofactor, PLP (Figure 1). Cancer cells may overexpress *PNPO* to bypass this bottleneck, rapidly increasing the PLP production necessary for accelerated proliferation and anabolic growth.

### 1.5. Mechanisms Underlying the Impact of Vitamin B6 on Cancer

PLP can impact cancer progression through multiple mechanisms arising from both its role as a cofactor and its antioxidant properties. Some of the mechanisms affecting genome integrity and gene expression are linked to vitamin B6 function in 1C-metabolism, which relies on the activity of three interconnected pathways: the folate cycle, the methionine cycle, and the trans-sulfuration pathway [78] (Figure 2). In the folate cycle, PLP serves as a cofactor for the enzyme SHMT, which converts serine into glycine and transfers the released 1C units to tetrahydrofolate (THF), giving rise to N5, N10-methylene THF. This compound is subsequently utilized by the thymidylate synthase (TS) enzyme for the biosynthesis of thymidylate (dTMP), an essential DNA precursor. Additionally, N5, N10-methylene THF can be reduced to methyl-THF to enter the methionine cycle, thereby regulating DNA and protein methylation patterns. In the trans-sulfuration pathway, PLP acts as a cofactor for enzymes involved in cysteine generation, a critical precursor for the synthesis of glutathione (GSH), one of the primary endogenous antioxidants.

**Figure 3:**
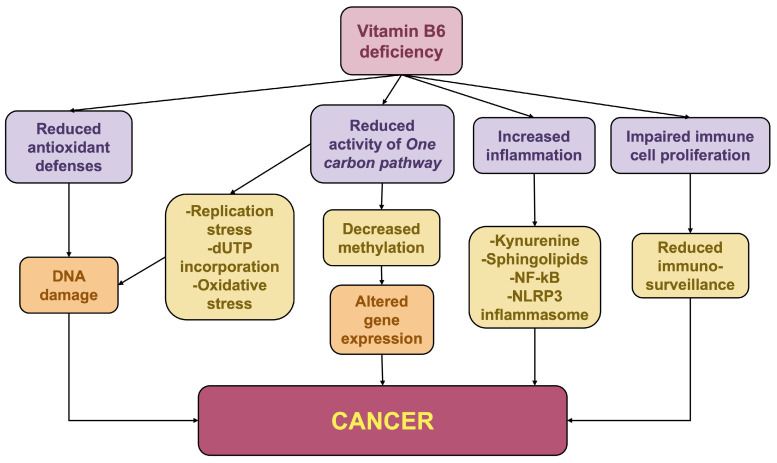
Vitamin B6 deficiency and cancer. Schematic representation of the main mechanisms linking vitamin B6 deficiency to cancer.

PLP deficiency has been shown to reduce SHMT activity in both human cells and *Drosophila* [79,80]. In the folate cycle, this reduction leads to decreased dTMP levels, creating a nucleotide imbalance that can trigger replication stress and stalled replication forks. Furthermore, an elevated dUTP/dTMP ratio can promote the misincorporation of uracil into DNA in place of thymidine; both mechanisms ultimately culminate in DNA double-strand breaks (DSBs) [81]. Beyond nucleotide imbalance, dTMP deprivation can further drive DSB formation by inducing the production of ROS [80,82,83] (Figure 3).

It is expected that PLP depletion also increases ROS by impairing the trans-sulfuration pathway, in which PLP acts as an essential cofactor for enzymes such as cystathionine beta-synthase (CBS) and cystathionine gamma-lyase (CSE), both critical for GSH synthesis. Furthermore, beyond its enzymatic role, vitamin B6 deficiency contributes to oxidative stress due to the loss of its direct antioxidant function, as PLP and its vitamers act as potent ROS scavengers capable of neutralizing superoxide radicals and lipid peroxides [16].

In addition to its role in nucleotide synthesis, SHMT is a key regulator of the methionine cycle. Reduced SHMT activity, due to the restricted availability of the PLP cofactor, is expected to diminish the pool of methyl groups, driving epigenetic dysregulation, a key hallmark of tumorigenesis [84] (Figure 3).

All the aforementioned mechanisms can induce mutations within oncogenes and tumor suppressor genes or alter their expression. DSBs that are not accurately repaired can lead to chromosome aberrations such as translocations, deletions, and gene amplifications [85]. These structural changes can result in the formation of oncogenic chimeric fusion proteins, the loss of critical anti-apoptotic functions, or the pathological overexpression of growth-promoting factors.

Beyond genomic instability, vitamin B6 deficiency can also contribute to malignancy by promoting chronic inflammation and compromising antitumor immunity (Figure 3). Vitamin B6 acts as a potent anti-inflammatory modulator [86]; accordingly, PLP levels are inversely correlated with markers of chronic inflammation [87]. While the underlying molecular mechanisms are not fully elucidated, relevant vitamin B6-dependent pathways include the kynurenine pathway and sphingosine 1-phosphate (S1P) metabolism [87,88]. Low B6 levels divert the kynurenine pathway towards the production of harmful metabolites that fuel inflammation. Moreover, vitamin B6 serves as an essential cofactor for sphingosine 1-phosphate lyase (SPL), the enzyme responsible for the irreversible degradation of S1P, a bioactive lipid that triggers pro-inflammatory pathways [88].

Additionally, vitamin B6 inhibits the NF-κB and NLRP3 inflammasome pathways, blocking the caspase-1 activation required to process pro-inflammatory cytokines into their mature forms [89,90].

PLP is indispensable for T-lymphocyte proliferation and differentiation, as well as for cytokine synthesis [14,91]. Specifically, vitamin B6 is essential for maintaining Natural Killer (NK) cell function in the context of antitumor immunity. This requirement can lead to metabolic competition between tumor cells and neighboring immune cells. For instance, in pancreatic ductal adenocarcinoma (PDAC), cancer cells restrict vitamin B6 availability, preventing NK cells from eliminating the tumor. This competition could potentially be counteracted by a synergistic combination of vitamin B6 supplementation and targeted blockade of vitamin B6-dependent one-carbon metabolism [92].

Recent evidence identifies the vitamin B6 vitamers PL and PLP as ligands for MR1 (major histocompatibility complex class I-related protein 1). Since MR1 facilitates antigen presentation to cancer-recognizing T cells, these findings suggest that fluctuations in vitamin B6 levels within tumor cells could influence the availability of PL and PLP as MR1 ligands, thereby modulating the immune recognition of cancer cells [93,94].

## 2. *Drosophila* to Model the Impact of Vitamin B6 Deficiency on Cancer

*Drosophila melanogaster* represents an exceptional model organism for addressing current challenges in vitamin B6 and cancer research. The *Drosophila* model offers significant advantages overcoming the limitations of both in vitro cell systems and epidemiological studies. Specifically, it enables investigations within a highly controlled environment, eliminating the confounding factors typically encountered in more complex mammalian systems. This model facilitates the establishment of clear cause-and-effect relationships, enabling a detailed exploration of underlying molecular mechanisms. This is further supported by the ease with which exogenous compounds can be administered and metabolized. Furthermore, *Drosophila* exhibits fewer toxic side-effects when treated with PLP inhibitors compared to mammalian models. Finally, its relatively simple karyotype allows for a precise analysis of chromosomal damage. A recent metabolomic analysis, conducted on larvae treated with the PLP antagonist 4-DP, revealed significant quantitative alterations, mainly attributable to the activity of PLP as a coenzyme in reactions affecting amino acid and sugar metabolism [95]. These findings validate the *Drosophila* model as a valuable tool to study the consequences of vitamin B6 alterations. In addition, they indicate that 4-DP supplementation is an effective experimental strategy to induce PLP deficiency in *Drosophila* functional studies.

### 2.1. Vitamin B6 and Genome Stability

Studies in *Drosophila* have established that PLP is essential for maintaining genomic integrity [96]. Specifically, mutations in *dPdxk*—the ortholog of the human *PDXK* gene (Figure 1)—lead to chromosome aberrations (CABs) in larval neuroblasts, which are fully rescued by PLP administration. Consistent with these findings, vitamin B6 antagonists, such as 4-DP, penicillamine, cycloserine, or isoniazid, induce high CAB frequencies in wild-type cells [96]. This role in genome maintenance is evolutionarily conserved; notably, the siRNA-mediated knockdown of human *PDXK*, as well as 4-DP treatment, triggers CABs and the formation of DNA repair foci also in human cells, recapitulating the phenotypes observed in *Drosophila* [96,97]. Additionally, the expression of a human wild-type *PDXK* transgene rescues the mutant phenotype within the *Drosophila dPdxk*^1^ mutant background [96]. Consistently, the expression of PDXK human variants with impaired catalytic activity fails to rescue the phenotype, leading to persistent chromosome damage in flies depleted of endogenous *dPdxk* [98]. In *Saccharomyces cerevisiae*, mutations in the pyridoxal kinase-encoding gene *BUD16* lead to gross chromosomal rearrangements, nucleotide imbalances, and increased sensitivity to hydroxyurea (HU), an inhibitor of ribonucleotide reductase [97]. However, the resulting DNA damage stems from an imbalanced nucleotide pool, which compromises DNA synthesis, rather than from uracil incorporation [97]. Conversely, although *Drosophila dPdxk*^1^ mutants likewise exhibit elevated dUTP levels compared to controls, they do not display comparable sensitivity to HU [96]. This suggests that the mechanisms behind DNA damage may diverge between these two species.

Further confirming the role of PLP in maintaining genomic integrity, the RNAi-mediated silencing of the *Drosophila sugarlethal (sgll)* gene, the *PNPO* ortholog (Figure 1), also resulted in chromosome and DNA damage [99]. The same effect was observed in neuroblasts from larvae in which the *sgll^PNPO^* gene was somatically mutagenized using the in vivo CRISPR-Cas9 system [99].

### 2.2. Effects of Vitamin B6 Deficiency in Drosophila Cancer Models

**Figure 4:**
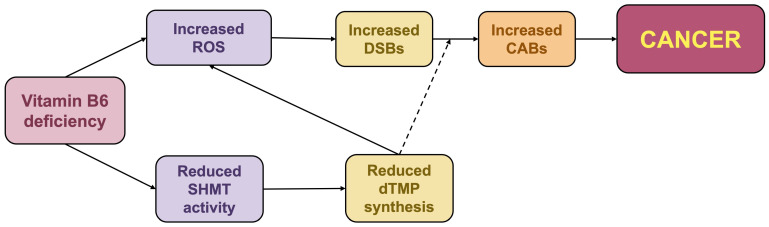
Schematic representation of the mechanisms underlying B6 deficiency-induced cancer in *Drosophila*. Reduced PLP levels lead to increased oxidative stress and the formation of DNA double-strand breaks (DSBs). Simultaneously, the impairment of the SHMT-mediated folate pathway results in dTMP depletion, which further fuels ROS production. This synergy exacerbates genomic instability and likely prevents correct DSB repair, promoting the accumulation of CABs and driving malignant transformation. The dashed line indicates the putative relationship (adapted from [80]).

It has been shown that vitamin B6 deficiency promotes cancer onset and progression in flies. In particular, treatment with PLP inhibitors can transform benign *Ras*^*V*12^ tumors into malignant forms [80]. In *Drosophila*, the expression of Ras^V12^ oncoprotein initially results in benign tumors; however, these tumors can undergo a malignant transformation when additional mutations or environmental stressors are introduced [100,101,102]. This transition closely mimics the multi-step nature of human tumorigenesis, in which a secondary hit, such as the loss of a tumor suppressor or a significant metabolic stressor, triggers invasions and metastases. Recent evidence has demonstrated that the transformation of *Ras*^*V*12^ tumors induced by PLP deficiency critically depends on ROS-mediated genomic instability. This oxidative stress derives from a synergistic effect: the loss of PLP’s direct antioxidant capacity and the disruption of its essential role as a cofactor for the SHMT enzyme (Figure 2) [80]. Indeed, when *Ras*^*V*12^ tumors are treated with PLP antagonists, such as 4-DP or ginkgotoxin (4′-O-methylpyridoxine), they not only display malignant hallmarks but also exhibit significant DNA and chromosome damage, together with oxidative stress and diminished SHMT activity. Treatment with antioxidants such as ascorbic acid (AA) or alpha-lipoic acid (ALA) fully rescues tumor phenotypes by abolishing both oxidative stress and DNA damage. Together, these findings indicate that ROS are the primary drivers of the genomic instability impacting tumor development. While SHMT depletion also causes a nucleotide imbalance that could promote DSBs, the finding that antioxidants alone can rescue the DNA damage suggests that genomic instability stems primarily from ROS. Intriguingly, dTMP supplementation also rescues tumor phenotypes, ROS levels, and DNA damage, demonstrating that a major consequence of dTMP depletion is the increased ROS production. The link between dTMP and ROS has also been reported by Ozer (2015) and Duo (2019), who attributed it to the increased activity of the NADPH Oxidase (NOX) enzyme induced by dTMP deprivation [82,83]. Consequently, a model with potential translational value has been proposed, in which PLP deficiency promotes malignancy by driving ROS-mediated genomic instability (Figure 4). This occurs through a synergistic mechanism: the loss of PLP’s antioxidant capacity and the impairment of SHMT activity, with dTMP depletion primarily amplifying ROS accumulation. In contrast, nucleotide imbalance serves as a secondary factor that exacerbates instability by impairing DSBs repair [80]. *Drosophila* has thus been instrumental not only in identifying genomic instability as the driver of *Ras*^*V*12^ tumor transformation, but also in clarifying the mechanisms by which SHMT depletion contributes to this process. The key role of ROS identified in *Drosophila* is further supported by mouse studies, where vitamin B6 supplementation significantly suppressed azoxymethane (AOM)-induced colon cancer by reducing oxidative stress markers [103].

PLP depletion also exacerbates *Ras^V12^csk^-/-^* tumors, characterized by *Ras*^*V*12^ overexpression combined with a homozygous null mutation in the *csk* gene, a negative regulator of Src kinase [104]. In this model, 4-DP feeding prevents *Ras^V12^csk^-/-^* tumor cells from undergoing apoptosis, thereby promoting their malignant proliferation (unpublished results).

Similarly, 4-DP accelerates the progression of *Ras^V12^Dlg^RNAi^* tumors by driving genomic instability. In this model, silencing the polarity gene *Disc large* (*Dlg*) transforms *Ras*^*V*12^ tumors into malignant forms [105] and the concomitant depletion of PLP further exacerbates the severity of these phenotypes. The specificity of the effect is underscored by the fact that silencing the *sgll^/PNPO^* gene recapitulates these phenotypes [80].

### 2.3. Gene–Nutrient Interaction Between PLP and SHMT in Cancer

Growing evidence indicates that gene–nutrient interactions between macro- and micronutrients and metabolic enzymes significantly impact cancer risk. A hallmark of this relationship is the one-carbon pathway, which regulates the transfer and exchange of methyl groups, utilizing various vitamins as essential cofactors. The classical paradigm of this interaction is represented by polymorphic variants of the Methylenetetrahydrofolate reductase (MTHFR) enzyme; when combined with reduced folate (B9) intake, these variants alter intracellular methylation levels and increase cancer risk [106]. Similarly, the synergy between genetic mutations that impair the enzymatic activity and the limited availability of the corresponding cofactor is expected to create a metabolic vulnerability that elevates oncogenic risk. To date, research examining the interplay between *SHMT1* polymorphisms and vitamin B6 levels regarding breast cancer risk has yielded promising results, although these findings remain limited by small sample size and necessitate further investigation [107].

Studies in *Drosophila* have significantly advanced our understanding of these interactions by providing critical mechanistic insights. RNAi-mediated depletion of *SHMT* triggers significant DNA damage and chromosome aberrations in *Drosophila* tissues [108,109]. Consistent with the finding that diminished PLP levels impair SHMT enzymatic activity, and given PLP’s additional antioxidant role, treatment with 4-DP has been shown to synergize with SHMT deficiency, further amplifying genomic instability in larval neuroblasts [108]. Intriguingly, this gene–nutrient interaction profoundly affects *Ras^V12^Dlg^RNAi^* tumors, where SHMT depletion alone is sufficient to induce genomic instability and ROS accumulation, thereby driving malignant progression. In the same cells, PLP depletion synergizes with SHMT depletion to strongly exacerbate DNA and chromosome damage; however, this synergy only marginally increases tumor growth and metastasis. This limited progression is attributed to the fact that extreme genomic damage triggers apoptosis, overriding the anti-apoptotic signals typically provided by the *Ras^V12^Dlg^RNAi^* background [109].

Translating these findings to humans, it can be hypothesized that, depending on the cellular context, this interaction places tumor cells at a critical crossroads: either they continue proliferating and thereby transmit substantial genomic damage, or they arrest proliferation and undergo apoptosis. Conversely, in human tumors where *SHMT* is overexpressed [110], this gene–nutrient interaction suggests that treatment with PLP inhibitors could synergize with therapies aimed at reducing SHMT activity, thereby enhancing apoptosis and inhibiting cancer growth [109]. However, considering the essential roles of PLP in global metabolism and cell physiology, as well as its crucial function in maintaining genome integrity, any therapeutic strategy involving PLP depletion should adopt a targeted precision-medicine approach to minimize systemic toxicity or mutagenic effects in normal cells.

Notably, tumor cells that rely on PLP availability for growth, and are therefore more sensitive to PLP depletion than non-tumor cells, may be particularly vulnerable. In such contexts, even a modest reduction in PLP levels could have a significant impact on cancer growth while exerting limited effects on normal tissues. Therefore, determining the optimal therapeutic dosage will be essential to avoid detrimental consequences associated with systemic PLP deficiency.

Alternatively, PLP depletion could be selectively targeted at cancer cells, for example through siRNA-mediated silencing of *PDXK* specifically delivered to tumor cells, thereby limiting off-target effects in healthy tissues.

### 2.4. PLP, Diabetes and Cancer Risk

In *Drosophila*, PLP deficiency—induced by either *dPdxk* mutations or 4-DP treatment- synergizes with dietary sugars (glucose, fructose, and sucrose), to enhance chromosome instability in larval neuroblasts [96].

Additionally, various *Drosophila* models of type 2 diabetes -generated through either the downregulation of conserved insulin signaling genes (*InR*, *chico*, and *Akt*^1^) or a high-sugar diet (HSD)—exhibit increased sensitivity to PLP depletion compared to non-diabetic controls. Specifically, upon 4-DP treatment, diabetic flies display DNA and chromosome damage levels approximately 2.5 to 3.5 fold higher than those observed in similarly treated non-diabetic flies [111,112]. In line with these findings, *Akt*^1^; *dPdxk*^1^ double mutants show a significantly higher frequency of CABs than single mutants, mirroring the hypersensitivity to 4-DP observed in diabetic backgrounds [111].

This heightened sensitivity to PLP depletion in a hyperglycemic context is particularly relevant as clinical evidence shows that individuals with diabetes often exhibit significantly reduced plasma PLP levels [20]. This indicates that vitamin B6 deficiency may exacerbate the already elevated cancer risk in diabetic patients [113].

Mechanistically, it has been shown that PLP depletion in diabetic flies drives the accumulation of AGEs, genotoxic compounds triggered by hyperglycemia. Supporting this model, the administration of alpha-lipoic acid, an AGE inhibitor, rescued both AGE levels and CAB frequency. Given that PLP naturally counteracts the AGE formation [7,111], it is conceivable that in 4-DP-treated diabetic cells, the loss of PLP-mediated protection promotes AGE accumulation, which in turn triggers ROS production, leading to DNA damage.

When extrapolated to humans, these findings suggest that low PLP levels may represent a critical mechanistic link between diabetes and cancer [113] (Zhou) by compromising DNA integrity through AGE-mediated oxidative stress.

### 2.5. Vitamin B6 Deficiency and Its Impact on Loss of Heterozygosity (LOH)

A crucial mechanism driving carcinogenesis is the loss of heterozygosity (LOH), a process in which cells carrying a heterozygous mutation in a tumor suppressor gene lose their wild-type allele. This transition leads to a state of hemizygosity for the mutation, which can effectively trigger malignant transformation. Notably, LOH creates distinct genetic differences between tumor and normal cells, providing opportunities for the development of novel selective cancer therapies [114].

Consistent with its genotoxic effects, PLP depletion induces LOH via mitotic recombination in flies heterozygous for mutations in the *warts (wts)* tumor suppressor gene [115]. Consequently, flies treated with 4-DP or ginkgotoxin develop epithelial tumors in multiple sites in the adult body with frequencies comparable to those induced by genotoxic agents such as benzo(e)pyrene, aflatoxin B2 (AFB2), 4-acetylaminofluorene (4-AAF), 4-(methylnitrosamino)-1-(3-pyridyl)-1-butanone (NNK), and oxoplatin tested within the same system [116]. When extrapolated to humans, these findings suggest that reduced PLP levels may trigger LOH in carriers of germline mutations such as *BRCA1/2* or *RB1*, thereby increasing their associated cancer risk. This highlights the role of vitamin B6 as a potential metabolic modifier of hereditary cancer predisposition.

## 3. Conclusions

**Figure 5:**
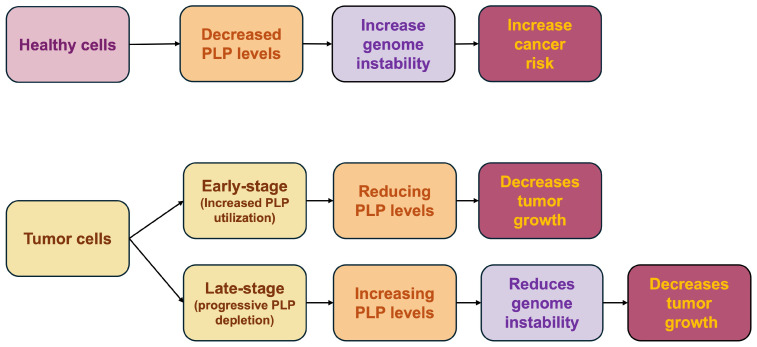
Schematic representation of PLP effect in different biological contexts. In healthy cells, reduced PLP levels increase cancer risk by promoting genome instability. In early-stage tumors, which rely on accelerated PLP consumption, PLP depletion reduces cancer growth. In late-stage tumors, PLP levels progressively diminish; in this context, PLP supplementation reduces genome instability and arrests tumor progression.

Clinical and experimental evidence reveals a complex metabolic landscape in which the response to vitamin B6 depends on both the stage of tumor progression and the specific nature of the malignancy. In non-transformed cells, PLP deficiency acts as a driver of oncogenesis by perturbing genomic stability. However, in established malignancies, the response is highly heterogeneous: while some cancer cells develop a hypermetabolic dependence on elevated PLP turnover to sustain rapid proliferation, others do not. As the disease progresses, PLP levels often decrease, creating a critical metabolic vulnerability that further impacts both genome stability and the immune response [22]. In this context, a favorable outcome may depend on increased expression of genes involved in PLP synthesis [70] (Figure 5).

Given the multifaceted role of vitamin B6 in oncogenesis, several biochemical mechanisms have been proposed to elucidate its impact on tumor development. Nonetheless, animal models remain indispensable for validating these processes in vivo and for uncovering novel regulatory pathways.

Mechanistic insights from *Drosophila* have demonstrated that vitamin B6 deficiency accelerates Ras-driven tumorigenesis by inducing genomic instability. This effect is mediated by increased oxidative stress resulting from the combined loss of the PLP’s antioxidant function and its role as a cofactor for SHMT. Studies in flies further support the hypothesis that PLP deficiency may exacerbate cancer risk in diabetic patients by promoting the accumulation of genotoxic AGEs. Furthermore, the fly model has elucidated the gene–nutrient interaction between PLP and *SHMT*, a link previously elusive in human research with significant therapeutic implications.

Ultimately, the discovery in flies that B6 deficiency can trigger tumorigenesis through loss of heterozygosity (LOH) provides a mechanistic bridge, directly linking micronutrient-dependent genome stability to cancer development.

In conclusion, this review highlights how *Drosophila* serves as a powerful discovery tool by bypassing the complexities of human epidemiology, providing the mechanistic insights needed to translate nutritional observations into concrete cancer prevention and therapeutic strategies. A better understanding of vitamin B6 metabolism in cancer may open new avenues for biomarker development and therapeutic targeting, as summarized in Box 1.

Box 1 **Clinical perspective.**

**BOX-1 Clinical Perspective**

**Potential implications for biomarker development.** Vitamin B6 metabolism offers opportunities for the identification of biomarkers relevant to cancer risk and treatment response. Plasma pyridoxal 5′-phosphate (PLP) remains the most widely used indicator of vitamin B6 status, while the PAr index (4-pyridoxic acid ratio) reflects vitamin B6 catabolism and inflammatory processes. Functional biomarkers such as the 3-hydroxykynurenine: xanthurenic acid (HK:XA) ratio provides insight into intracellular vitamin B6 availability. Emerging candidates include altered expression of enzymes involved in vitamin B6 metabolism (e.g., *PDXK* or *PNPO*) and perturbations of PLP-dependent pathways, particularly those linked to one-carbon metabolism.

**Nutritional interventions.** Adequate vitamin B6 intake can generally be achieved through a varied diet and supports genome stability and normal cellular metabolism. Supplementation may be beneficial in cases of deficiency to decrease cancer risk. Routine high-dose supplementation is not currently recommended.

**Combination strategies with chemotherapy.** Modulation of vitamin B6 metabolism may influence tumor sensitivity to anticancer therapies. Preclinical evidence indicates that PDXK-dependent activation of vitamin B6 enhances the cytotoxic activity of several chemotherapeutic agents [70].

**Targeted therapies.** Targeting PLP-dependent metabolic pathways may represent a promising strategy to improve therapeutic responses. Potential approaches include vitamin B6 supplementation combined with inhibition of vitamin B6-dependent one-carbon metabolism in pancreatic ductal adenocarcinoma (PDAC) [92], as well as the use of SHMT inhibitors together with PLP inhibitors or with the silencing of genes involved in PLP metabolism in cancers overexpressing SHMT [106]. The development of inhibitors targeting enzymes involved in PLP metabolism may represent an additional therapeutic opportunity [117].

## Data Availability

No new data were created or analyzed in this study.
